# Antibiofilm and Antivirulence Activities of 6-Gingerol and 6-Shogaol Against *Candida albicans* Due to Hyphal Inhibition

**DOI:** 10.3389/fcimb.2018.00299

**Published:** 2018-08-28

**Authors:** Jin-Hyung Lee, Yong-Guy Kim, Pilju Choi, Jungyeob Ham, Jae Gyu Park, Jintae Lee

**Affiliations:** ^1^School of Chemical Engineering, Yeungnam University, Gyeongsan, South Korea; ^2^Natural Products Research Institute, Korea Institute of Science and Technology, Gangneung, South Korea; ^3^Advanced Bio Convergence Center, Pohang Technopark Foundation, Pohang, South Korea

**Keywords:** antivirulence, biofilm, *C. albicans*, gingerol, hyphae, shogaol

## Abstract

*Candida albicans* is an opportunistic pathogen and responsible for candidiasis. *C. albicans* readily forms biofilms on various biotic and abiotic surfaces, and these biofilms can cause local and systemic infections. *C. albicans* biofilms are more resistant than its free yeast to antifungal agents and less affected by host immune responses. Transition of yeast cells to hyphal cells is required for biofilm formation and is believed to be a crucial virulence factor. In this study, six components of ginger were investigated for antibiofilm and antivirulence activities against a fluconazole-resistant *C. albicans* strain. It was found 6-gingerol, 8-gingerol, and 6-shogaol effectively inhibited biofilm formation. In particular, 6-shogaol at 10 μg/ml significantly reduced *C. albicans* biofilm formation but had no effect on planktonic cell growth. Also, 6-gingerol and 6-shogaol inhibited hyphal growth in embedded colonies and free-living planktonic cells, and prevented cell aggregation. Furthermore, 6-gingerol and 6-shogaol reduced *C. albicans* virulence in a nematode infection model without causing toxicity at the tested concentrations. Transcriptomic analysis using RNA-seq and qRT-PCR showed 6-gingerol and 6-shogaol induced several transporters (*CDR1, CDR2*, and *RTA3*), but repressed the expressions of several hypha/biofilm related genes (*ECE1* and *HWP1*), which supported observed phenotypic changes. These results highlight the antibiofilm and antivirulence activities of the ginger components, 6-gingerol and 6-shogaol, against a drug resistant *C. albicans* strain.

## Introduction

*Candida albicans* is an opportunistic pathogen normally present on skin and mucous membranes, such as, those of the vagina, mouth, and rectum. *C. albicans* colonizes host tissues and various indwelling medical devices (Ramage et al., 2005; Sardi et al., 2013) and readily develops biofilms on biotic and abiotic surfaces that are intrinsically resistant to conventional antifungal therapeutics and the host immune system (Nobile et al., 2006b). *C. albicans* can grow as oval budding yeasts, pseudohyphae, or true hyphae. For biofilm development, yeast cells initially attach to a surface, and this is then followed by germ tube formation and hyphal transition, and mature biofilms are typically formed within 24 h (Nobile et al., 2006b). The transition of yeast cells to hyphal cells appears to regulate biofilm maturation, and hyphal transition is considered a crucial virulence factor in *Candida* infections (Carradori et al., 2016). Also, many clinical isolates of *C. albicans* exhibit drug resistance against commercial antifungals, such as, azoles and polyenes, which are used to treat candidiasis (Tobudic et al., 2010; Taff et al., 2013; Sandai et al., 2016). Hence, novel antivirulence drugs not prone to the development of antifungal resistance, are required to eradicate *C. albicans* biofilms and virulence.

Phytochemicals are important sources for antimicrobial and antibiofilm agents against drug resistant microorganisms (Nascimento et al., 2000). Recently, several studies have demonstrated ginger components have antibiofilm activities against pathogenic bacteria, such as, ginger water extract against *Pseudomonas aeruginosa* (Kim and Park, 2013) and against *Salmonella Typhimurium* and *Escherichia coli* (Khiralla, 2015), and zingerone (Kumar et al., 2013), raffinose (Kim et al., 2016a), 6-gingerol (Kim et al., 2015), and 6- and 8-gingerol analogs (Choi et al., 2017) against *P. aeruginosa*. However, the antibiofilm activities of ginger components have not been studied in any yeast species.

In this study, the antibiofilm activities of six ginger components, namely, 6-gingerol, 8-gingerol, 10-gingerol, 6-shogaol, 8-shogaol, and 10-shogaol, were initially investigated against antifungal-resistant *C. albicans* strain. Two active compounds 6-gingerol and 6-shogaol were further evaluated with respect to hyphal and virulence inhibition. Scanning electron microscopy (SEM) and confocal laser scanning microscopy (CLSM) were used to investigate the effects of 6-gingerol and 6-shogaol on morphological changes, biofilm formation, and on the hyphal growth of *C. albicans*. The molecular basis of the alterations in *C. albicans* physiology upon exposure to 6-gingerol and 6-shogaol was also investigated using RNA-seq and qRT-PCR. In addition, an *in vivo Caenorhabditis elegans* model was used to confirm the antivirulence efficacies of 6-gingerol and 6-shogaol. This is the first report to be issued regarding the use of 6-gingerol or 6-shogaol to inhibit *C. albicans* biofilm formation and hyphal formation and to reduce the virulence of this pathogen.

## Materials and methods

### Strains and medium

In this study, we used fluconazole resistant *C. albicans* strain DAY185 (minimum inhibitory concentration >1,024 μg/ml). *C. albicans* was maintained in potato dextrose agar (PDA) or potato dextrose broth (PDB). The gingerols and shogaols (6-gingerol, 8-gingerol, 10-gingerol, 6-shogaol, 8-shogaol, and 10-shogaol) used in this study were purchased from Sigma-Aldrich (St. Louis, USA) and dissolved in dimethyl sulfoxide (DMSO). DMSO was used as a negative control for all experiments and the concentration of DMSO in media did not exceed 0.1% (vol/vol), which did not affect the antibiofilm or antivirulence activities. Cell growths and turbidities were measured using spectrophotometer (UV-160, Shimadzu, Japan) at 620 nm.

### Assays for biofilm formation

*Candida* biofilms were developed on 96-well polystyrene plates, as previously reported (Lee et al., 2011). Briefly, a 2-day single colony was inoculated into 25 ml of PDB and incubated overnight at 37°C. Overnight cultures at an initial turbidity of 0.1 at 600 nm were then inoculated into PDB (final volume 300 μl) with or without a gingerol or a shogaol, and incubated for 24 h without shaking at 37°C. Biofilm cells that adhered to the 96-well plates were stained with 0.1% crystal violet (Sigma-Aldrich, St. Louis, USA) for 20 min, washed repeatedly with sterile distilled water, and resuspended in 95% ethanol. Plates were read at 570 nm and results are presented as the averages of at least six repetitions.

### Confocal laser scanning microscopy assay of biofilm formation

*C. albicans* biofilms were grown on 96-well plates with or without 6-gingerol or 6-shogaol without shaking for 24 h. Planktonic cells were then removed by washing with water three times, and biofilms were stained with carboxyfluorescein diacetate succinimidyl ester (a minimally fluorescent lipophile; Invitrogen, Molecular Probes, Inc, Eugene, USA) (Lee et al., 2016). Plate bases were then visualized using an (a 488 nm) Ar laser (emission 500 to 550 nm) under a confocal laser microscope (Nikon Eclipse Ti, Tokyo), and COMSTAT biofilm software (Heydorn et al., 2000) was then used to calculate biovolumes (μm^3^ μm^−2^), mean biofilm thicknesses (μm), and substratum coverages (%). Two independent cultures were performed under each experimental condition and at least 10 random positions were assayed.

### Observation of *C. albicans* colony morphologies on solid media

A freshly prepared glycerol stock of *C. albicans* was used to streak on PDA plates supplemented with and without 6-gingerol or 6-shogaol. Plates were then incubated for 7 days at 37°C and temporal changes in colony morphologies were observed using an iRiS™ Digital Cell Imaging System (Logos Bio Systems, Korea).

### Hyphal assay in liquid media

Cell aggregation was analyzed as previously described (Zelante et al., 2012). Briefly, *C. albicans* cells were inoculated into 2 ml of PDB medium or RPMI-1640 medium at density of 10^5^ CFU/ml in 14 ml test tubes with or without 6-gingerol or 6-shogaol and incubated at 37°C for 24 h with shaking at 250 rpm. Cell cultures (2 ml) were then transferred into glass-bottom dishes and observed. Aggregated cells were visualized in bright field using the iRiS^TM^ Digital Cell Imaging System (Logos Bio Systems, Korea) at a magnification of 4x. At least, four independent experiments were conducted.

### Microscopic imaging of hyphal formation

Scanning electron microscopy (SEM) was used to observe the morphologies of biofilm cells attached to a nylon membrane, as previously described (Kim et al., 2016b). Briefly, a nylon membrane was cut into 0.5 × 0.5 cm pieces and placed in 96-well plates containing *C. albicans* grown with or without 6-gingerol or 6-shogaol and incubated for 24 h at 37°C. Cells that adhered to the nylon membrane were fixed with glutaraldehyde (2.5%) and formaldehyde (2%) for 24 h and then post-fixed using osmium, dehydrated with an ethanol series (50, 70, 80, 90, 95, and 100%), and isoamyl acetate. After critical-point drying, cells were examined and imaged using a S-4100 scanning electron microscope (Hitachi, Japan) at a voltage of 15 kV.

### RNA isolation for RNA-Seq and quantitative real-time PCR (qRT-PCR)

For transcriptomic analyses, 25 ml of *C. albicans* at an initial turbidity of 0.1 at OD_600_ was inoculated into PDB in 250 ml Erlenmeyer flasks and incubated for 4 h at 37°C with agitation (250 rpm) in the presence or absence of 6-gingerol (50 μg/ml) or 6-shogaol (10 μg/ml). To prevent RNA degradation, RNase inhibitor (RNAlater, Ambion, TX, USA) was added to cells. Total RNA was isolated using a hot acidic phenol method (Amin-ul Mannan et al., 2009), and RNA was purified using a Qiagen RNeasy mini Kit (Valencia, CA, USA).

### RNA-Seq and RNA library preparation and sequencing

For RNA-Seq, a RNA library was constructed using the SMARTer Stranded RNA-Seq Kit (Clontech Laboratories, Inc., USA). Briefly, 2 μg of total RNA was incubated with magnetic beads decorated with oligo-dT and then RNAs, other than mRNA, were removed using washing solution. Library production was initiated by the random hybridization of starter/stopper heterodimers to poly(A) RNA bound to the magnetic beads. These starter/stopper heterodimers contained Illumina-compatible linker sequences. A single-tube reverse transcription and ligation reaction extended the starter to the next hybridized heterodimer, where the newly-synthesized cDNA insert was ligated to the stopper. Second strand synthesis was performed to release the library from the beads, and the library was then amplified. Barcodes were introduced when the library was amplified. High-throughput sequencing was performed by paired-end 100 sequencing using HiSeq 2500 (Illumina, Inc., USA).

### RNA-Seq data analysis

mRNA-Seq reads were mapped using TopHat software (Trapnell et al., 2009) in order to obtain the alignment file. Differentially expressed genes were identified based on counts from unique and multiple alignments using Bedtools (Quinlan and Hall, 2010). RT (Read Count) data were processed by Quantile normalization using Bioconductor (Gentleman et al., 2004). The alignment files also were used to assemble transcripts, estimate their abundances, and to detect the differential expressions of genes or isoforms using Cufflinks. FPKM (fragments per kilobase of exon per million fragments) was used to determine the expression levels of gene regions. Gene classification was based on the results of searches performed using DAVID (http://david.abcc.ncifcrf.gov/). The RNA-seq data were deposited at NCBI Gene Expression Omnibus and are accessible through accession number GSE117201. Differentially expressed gene study was analyzed with the DEG analysis method in ExDEGA (Excel based Differentially Expressed Gene Analysis) tool and classified by biological processes. Gene ontology analysis was performed at QuickGO (www.ebi.ac.uk/QuickGO/). KEGG (Kyoto Encyclopedia of Genes and Genomes) pathway analyses of the RNA-seq data were performed with the KEGG Mapper tool (http://www.genome.jp/kegg/tool/map_pathway2.html).

### qRT-PCR

qRT-PCR was used to determine the expressions of hyphae-related genes (*ALS1, ALS3, ECE1, ECM38, EED1 EFG1, HYR1, HWP1, RBT1, SAP4*, and *UME6*). The specific primers and housekeeping gene (*RDN18*) used for qRT-PCR are listed in Supplementary Table S2. The qRT-PCR method used was as described by Kim et al. (2016b), and performed using SYBR Green master mix (Applied Biosystems, Foster City, USA) and an ABI StepOne Real-Time PCR System (Applied Biosystems). At least two independent cultures were used.

### Antivirulence and toxicity assays using the *Caenorhabditis elegans* model

For the antivirulence assay, we used *C. elegans* strain *fer-15 (b26)*; *fem-1 (hc17)*, as previously described (Manoharan et al., 2017a). Briefly, synchronized adult worms were fed on *C. albicans* lawns for 4 h at 25°C and then collected after washing three times with M9 buffer. Approximately 30 worms were then added to each well of 96-well plates containing PDB medium (300 μl) with or without 6-gingerol (10 or 50 μg/ml) or 6-shogaol (10 or 50 μg/ml). Assay plates were then incubated for 4 days at 25°C without shaking. For toxicity assays, 30 non-infected worms were pipetted into single wells of a 96-well dish containing M9 buffer and solutions of 6-gingerol or 6-shogaol were added to final concentrations of (0, 100, 200, or 500 μg/ml) without *C. albicans*. Plates were then incubated for 4 days at 25°C without shaking. Three independent experiments were performed in triplicate. Results are expressed as percentages of live worms (survival), as determined by responses to platinum wire touching after incubation for 4 days. Observations were made using an iRiS™ Digital Cell Imaging System (Logos Bio Systems, Korea).

### Statistical analysis

Replication numbers for assays are provided above and results are expressed as means ± standard deviations. The statistical analysis was performed by one-way ANOVA followed by Dunnett's test using SPSS version 23 (SPSS Inc., Chicago, IL, USA). *P*-values of < 0.05 were regarded significant.

## Results

### Inhibitory effects of gingerols and shogaols on *C. albicans* biofilm formation

**Figure 1:**
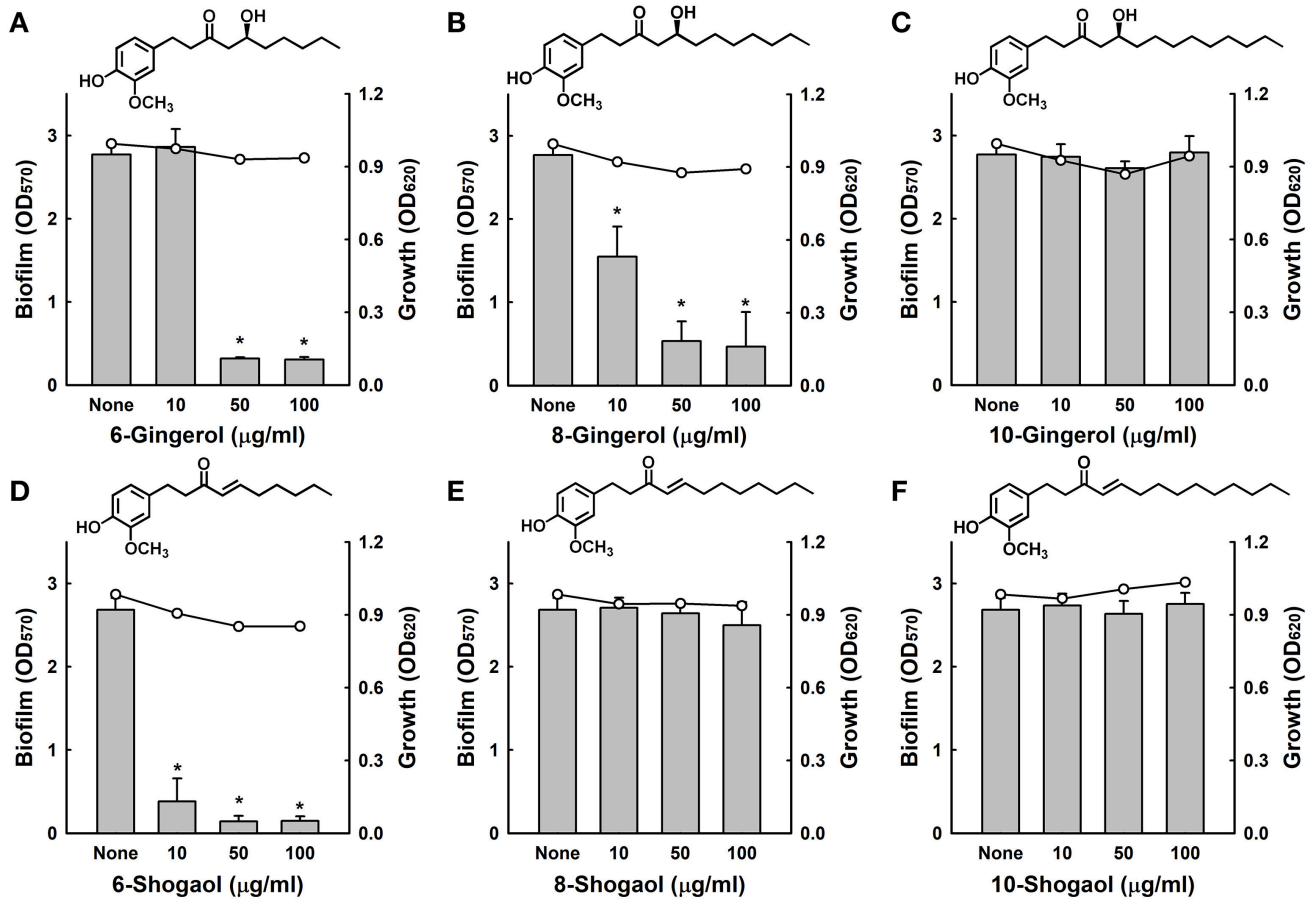
Antibiofilm activities of gingerols and shogaols against *C. albicans*. The antibiofilm activities of three gingerols **(A–C)** and three shogaols **(D–F)** against *C. albicans* DAY185 in PDB medium were determined after culture for 24 h. Bars indicate biofilm formation and lines indicate planktonic cell growth. The chemical structures of gingerols and shogaols are shown. *P < 0.05 *vs*. non-treated controls. None; non-treated control.

Initially, we investigated whether three gingerols (6-gingerol, 8-gingerol, and 10-gingerol) and three shogaols (6-shogaol, 8-shogaol, and 10-shogaol) affect biofilm formation by fluconazole-resistant *C. albicans* DAY185, cell growth was also measured in the presence of these agents. Of the six compounds, 6-gingerol, 8-gingerol, and 6-shogaol significantly reduced biofilm formation at concentrations of 10, 50, and 100 μg/ml, while 10-gingerol, 8-shogaol, and 10-shogaol at 100 μg/ml had no effect (Figure 1). In particular, 6-shogaol most significantly inhibited biofilm formation in a dose-dependent manner (Figure 1D). Specifically, 6-shogaol inhibited biofilm formation by 85, 94, and 94% at concentrations of 10, 50, and 100 μg/ml, respectively (Figure 1D). In addition, 6-gingerol and 8-gingerol at 50 μg/ml inhibited biofilm formation by 88 and 80%, respectively (Figures 1A,B). It appeared the antibiofilm activities of gingerols and shogaols were related to the number of carbon side chains as larger carbon side chain numbers appeared to decrease antibiofilm activity in 10-gingerol, 8-shogaol, and 10-shogaol (Figures 1C,E,F). Notably, none of the three gingerols or three shogaols at concentrations up to 100 μg/ml inhibited the planktonic cell growth of *C. albicans* (Figures 1A–F).

The antifungal activities of 6-gingerol and 6-shogaol were investigated by measuring minimum inhibitory concentrations (MIC), and for 6-gingerol and 6-shogaol MICs were 1000 μg/ml and > 2000 μg/ml, respectively, against *C. albicans* DAY185. These results support the notion that biofilm formation by *C. albicans* was effectively inhibited by the antibiofilm activities of 6-gingerol and 6-shogaol and not by their fungicidal activities. Furthermore, the observed biofilm inhibition in the absence of any effect on planktonic cell growth suggests that unlike conventional fungicides, 6-gingerol and 6-shogaol may less prone to the development of drug resistance.

**Figure 2:**
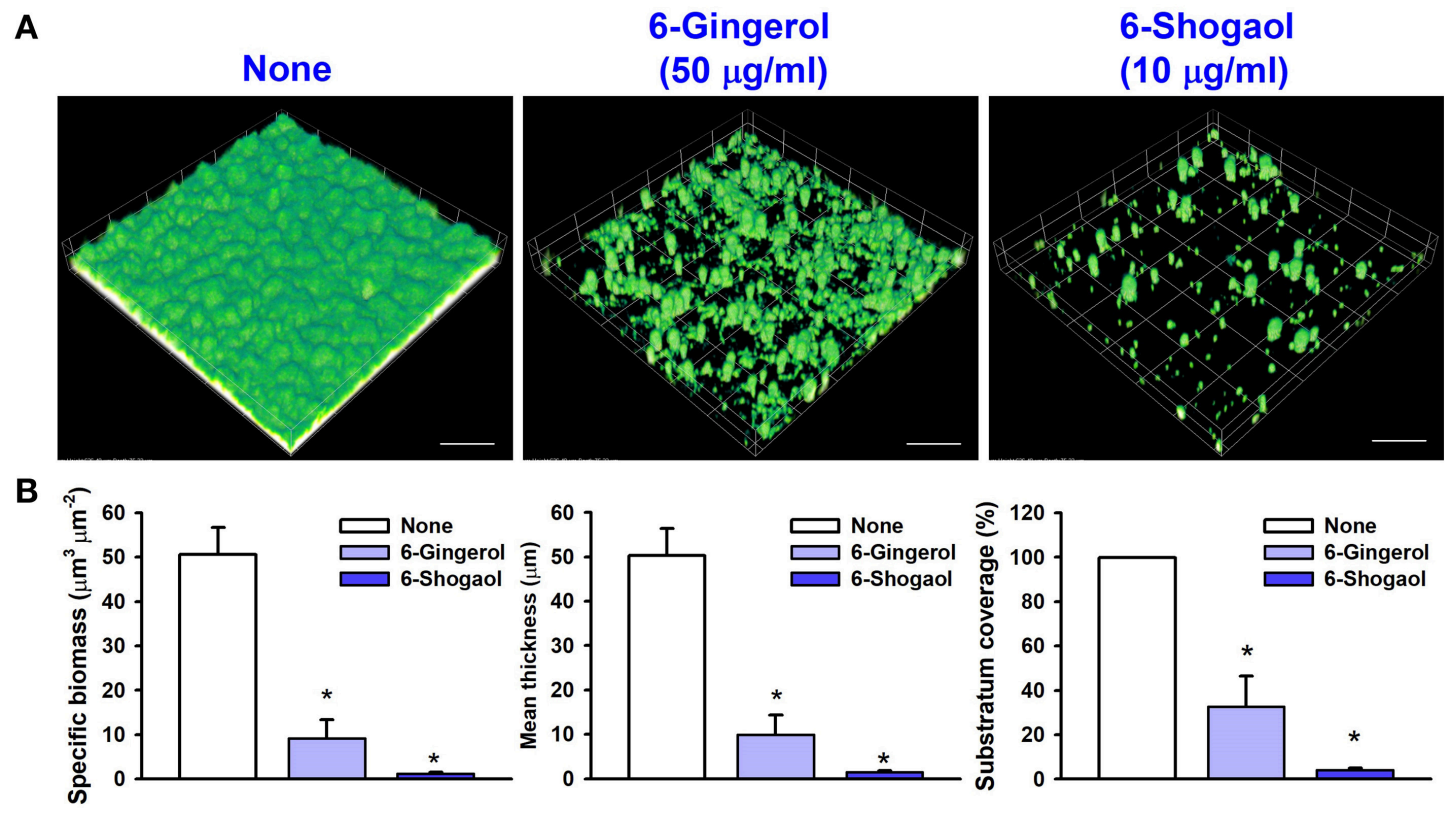
Microscopic observations of the inhibitory effects of 6-gingerol and 6-shogaol on biofilms. Biofilm formation by *C. albicans* on polystyrene plates was observed in the presence of 6-gingerol at 50 μg/ml or 6-shogaol at 10 μg/ml by confocal laser microscopy **(A)**. Scale bars represent 100 μm. Biofilm formation was quantified by using COMSTAT **(B)**. *P < 0.05 *vs*. non-treated controls. None; non-treated control.

Confocal laser microscope images showed that *C. albicans* formed dense biofilms in non-treated control samples, and that in the presence of 6-gingerol or 6-shogaol biofilm cellular densities and thicknesses were dramatically reduced (Figure 2A). Biofilm reduction was further confirmed by COMSTAT analysis, which showed 6-gingerol at 50 μg/ml and 6-shogaol at 10 μg/ml significantly reduced biofilm biomass, average thickness, and substrate coverage (Figure 2B). Specifically, biofilm biomass, thickness, and substrate coverage were reduced by 6-shogaol by more than 95% vs. the untreated control.

### 6-Gingerol and 6-Shogaol inhibited hyphal growth and cell aggregation

**Figure 3:**
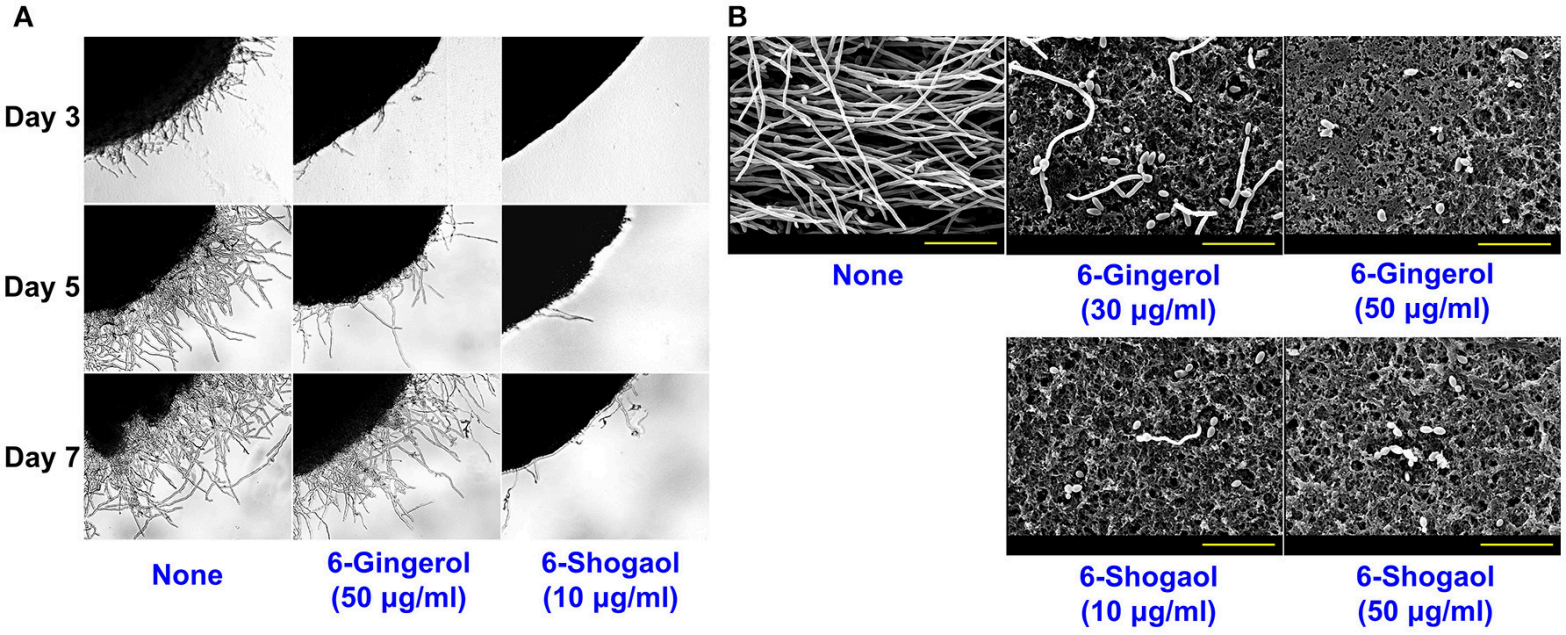
Effects of 6-gingerol and 6-shogaol on the hyphal morphogenesis of *C. albicans*. *C. albicans* morphology on solid media **(A)**. *C. albicans* was streaked on PDA solid plates in the absence or presence of 6-gingerol or 6-shogaol. Colony morphologies were observed during incubation for 7 days at 37°C. Inhibitions of hyphal growths by 6-gingerol or 6-shogaol in *C. albicans* biofilms were visualized by SEM **(B)**. The scale bar represents 30 μm. None; non-treated control.

To examine the effects of 6-gingerol and 6-shogaol on *C. albicans* morphology, a temporal observation of *C. albicans* colonies on potato dextrose agar (PDA) was performed and scanning electron microscope (SEM) was also used. Whereas hyphal protrusions from colonies of untreated *C. albicans* were observed after 3 days of incubation, in the presence of 6-shogaol at 10 μg/ml suppressed hyphal protrusions for 7 days (Figure 3A). Furthermore, 6-shogaol at 10 μg/ml was found to more effectively suppress hyphal protrusions than 6-gingerol at 50 μg/ml. SEM analysis also confirmed 6-gingerol and 6-shogaol substantially suppressed hyphal formation. As shown in Figure 3B, non-treated biofilms consisted predominately of hyphae and few pseudohyphae, where biofilms grown in the presence of 6-gingerol or 6-shogaol had shorter hyphae and more yeast cells.

**Figure 4:**
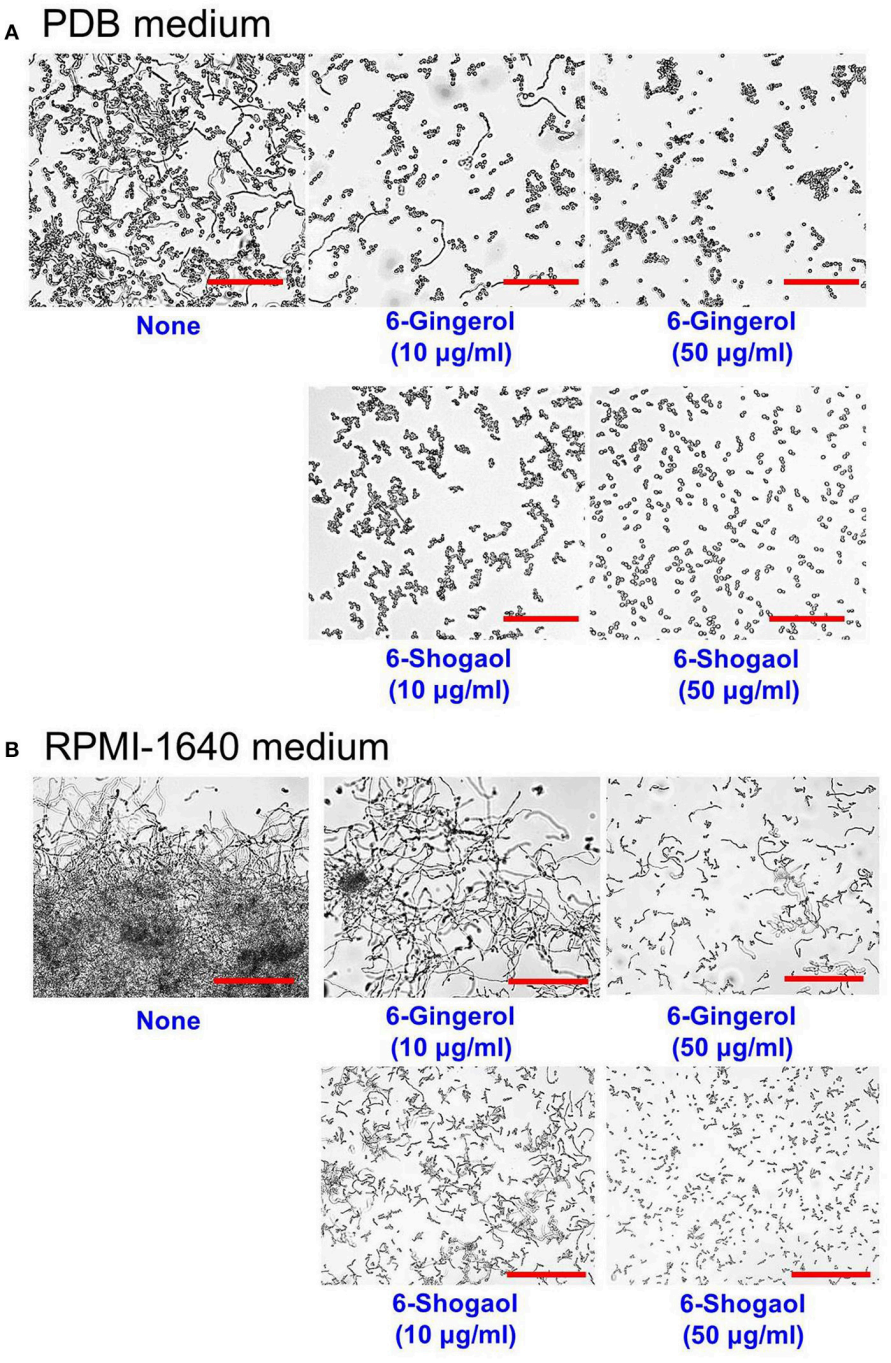
Inhibitions of hyphal filamentation and aggregation by 6-gingerol and 6-shogaol in liquid medium. Inhibitions of hyphal filamentation in PDB medium **(A)** and in RPMI medium **(B)**. *C. albicans* cells were grown for 24 h in PDB medium or RPMI-1640 medium in the absence or presence of 6-gingerol or 6-shogaol. Hyphae were visualized after incubation for 24 h. The scale bar represents 200 μm. None; non-treated control.

It is generally believed yeast-to-hypha-transition and cell aggregation are prerequisites of biofilm development by *C. albicans* (Chandra et al., 2001). In liquid potato dextrose broth (PDB) medium, hyphal inhibition was evident in the presence of 6-gingerol or 6-shogaol and more marked in the presence of 6-shogaol (Figure 4A). Another hyphal assay was performed using RPMI-1640 medium, which promotes hyphal formation (Kucharikova et al., 2011). After incubation for 24 h, mostly hyphae and large cell aggregations entangled by hyphae were observed in the control sample whereas treatment with 6-gingerol or 6-shogaol resulted in much smaller cell aggregations in a dose-dependent manner (Figure 4B). Furthermore, hyphal and cell aggregation results were in-line with the observed antibiofilm activities of 6-gingerol and 6-shogaol. Taken together, these results show 6-gingerol and 6-shogaol both potently inhibited hyphal formation and cell aggregation, and thus, suggest these two agents reduced biofilm formation by *C. albicans*.

### Differential gene expressions by 6-Gingerol and 6-Shogaol

The molecular bases of the effects of 6-gingerol or 6-shogaol on biofilm formation and hyphal growth were investigated by RNA-seq and qRT-PCR. RNA-seq was first used to determine differential gene expressions in untreated sample and treated samples. Genes differentially expressed by at least 2-fold were selected and sorted into four functional categories including biofilm and hyphae-related genes or virulence-related genes (Supplementary Table S1). Overall, expression trends were similar after treatment with 6-gingerol at 50 μg/ml or 6-shogaol at 10 μg/ml. However, in view of the concentrations used 6-shogaol clearly had a greater effect than 6-gingerol. The addition of 6-gingerol significantly altered the expressions of 125 genes by more than 2-fold; 37 genes were up-regulated and 88 genes were down-regulated. Similarly, the addition of 6-shogaol significantly altered the expressions of 78 genes; 29 genes were up-regulated and 49 genes were down-regulated.

**Figure 5:**
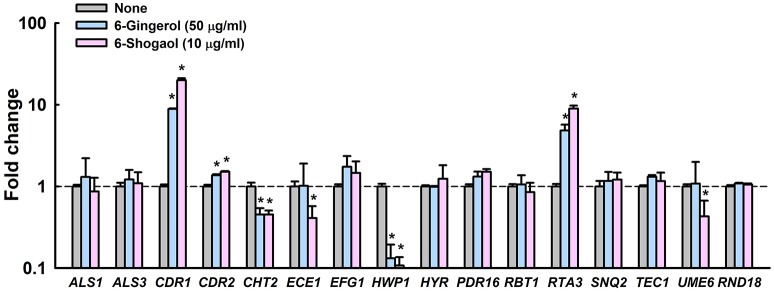
Transcriptional profiles of *C. albicans* cells treated with or without 6-gingerol or 6-shogaol. *C. albicans* was cultivated with or without 6-gingerol at 50 μg/ml or 6-shogaol at 10 μg/ml for 4 h with shaking at 250 rpm. Transcriptional profiles were obtained by qRT-PCR. Fold changes represent changes in the transcriptions of treated vs. untreated *C. albicans*. The experiment was performed in duplicate (six qRT-PCR reactions were performed per gene). *P < 0.05 *vs*. non-treated controls (None).

Notably, these expressional changes involved various biofilm- and hypha-related genes (Supplementary Table S1). Specifically, *HWP1* (hyphal cell wall protein, also known as *ECE2*) and *ECE1* (hypha-specific protein) were repressed by 6-gingerol or 6-shogaol by more than 7- and 2-fold, respectively, and *CDR1* and *CDR2* (multidrug transporter) and *RTA3* (lipid-translocating exporter) were up-regulated by 6-gingerol or 6-shogaol more than 4-fold. qRT-PCR was used to confirm gene expressional changes of highly differentially expressed loci in the 6-gingerol and 6-shogaol RNA-seq experiments. qRT-PCR for 15 selected genes showed differential changes in expression that generally concurred with RNA-seq assay results (Figure 5). For 6-shogaol experiment, RNA-seq and qRT-PCR showed the genes were repressed to similar extents, i.e., 10-fold vs. 20-fold for *CDR1*, 3-fold vs. 2-fold for *CHT2*, 12-fold vs. 9-fold for *HWP1*, 6-fold vs. 9-fold for *RTA3*, respectively. Similarly, 6-gingerol down-regulated the expression of *HWP1* and *CHT2*, and upregulated *CDR1* and *RTA3*. Nevertheless, the expressions of other biofilm and hyphae-related genes (*ALS1, ALS3, EFG1, HYR, PDR16, RBT1, SNQ2, TEC1*, and *UME6*) were unaffected by 6-gingerol or 6-shogaol. Taken together, RNA-seq and qRT-PCR results showed that 6-gingerol and 6-shogaol significantly altered the expressions of some hypha-specific (*HWP1* and *ECE1*), biofilm-related (*HWP1* and *RTA3*) and multidrug transporter (*CDR1* and *CDR2*) related genes.

### 6-Gingerol and 6-Shogaol rescued nematodes infected with *C. albicans*

**Figure 6:**
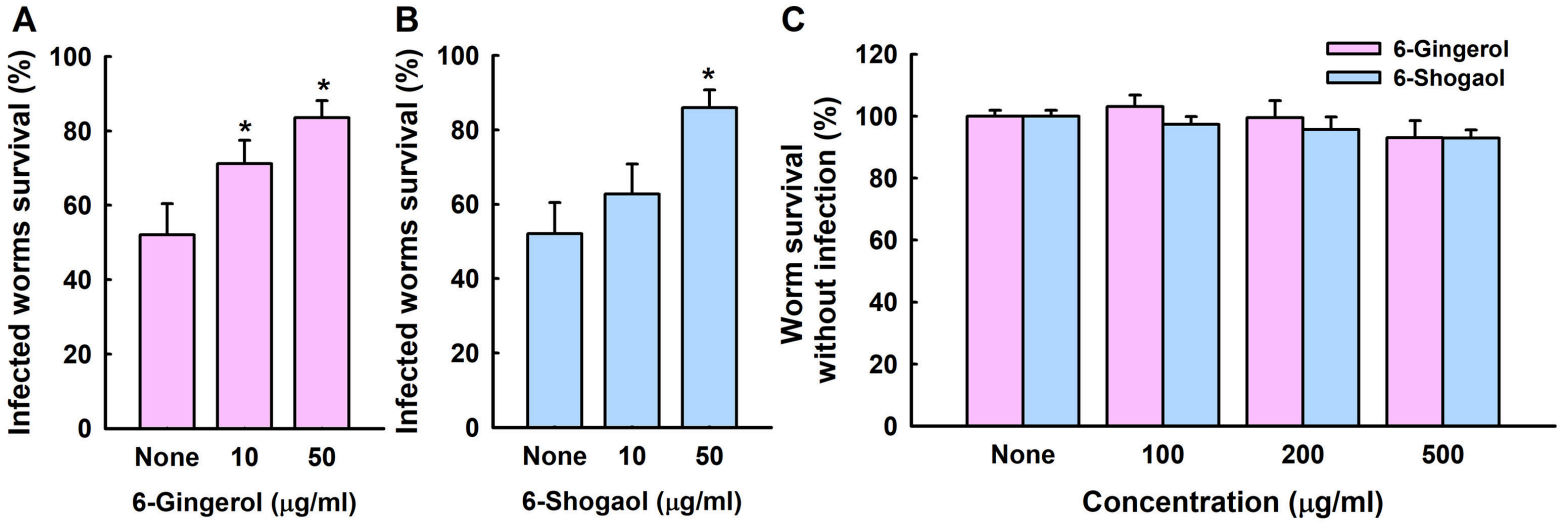
Effects of 6-gingerol and 6-shogaol on *C. albicans* infected *C. elegans*. Nematode survival after exposure to *C. albicans* for 4 days in the presence of 6-gingerol **(A)** or 6-shogaol **(B)**. The toxicities of 6-gingerol and 6-shogaol were determined by treating non-infected nematodes for 4 days **(C)**. None indicates non-treated controls. Worm survival was determined based on movement. *p < 0.05 *vs*. non-treated controls.

We examined whether 6-gingerol or 6-shogaol could affect *C. albicans* virulence in a *Caenorhabditis elegans* nematode model, which is an accepted alternative to mammalian models (Tampakakis et al., 2008). *C. albicans* infection caused 45% *C. elegans* fatality in 4 days. However, > 80% of nematodes survived in the presence of 6-gingerol or 6-shogaol at 50 μg/ml (Figures 6A,B). To investigate the chemical toxicities of 6-gingerol and 6-shogaol, non-infected nematodes were exposed to different concentrations of the two agents. We found 6-gingerol and 6-shogaol at concentrations up to 500 μg/ml were not toxic to *C. elegans* (Figure 6C). These results show that both 6-gingerol and 6-shogaol effectively promoted the survival of infected nematodes and that they had no toxic effects on the nematode.

## Discussion

Current study shows for the first time that the ginger components 6-gingerol and 6-shogaol reduce biofilm formation by a drug-resistant *C. albicans* strain by inhibiting hyphae growth and cell aggregation, and reduced fungal virulence.

Ginger (*Zingiber officinale* (L.) Rosc) has been used as a spice and herbal medicine for over 2000 years. Its roots and extracts contain polyphenol compounds, such as, gingerols, shogaols, paradols, gingerdiols, and zingerone, which have considerable antioxidant activity (Si et al., 2018). Fresh ginger contains about 4% of 6-gingerol by weight but almost no 6-shogaol. However, 6-shogaol is easily produced by dehydrating 6-gingerol using drying processes (Chen et al., 1986; Jolad et al., 2004). 6-Gingerol and 6-shogaol have been reported to be effective treatments for metabolic syndrome, cardiovascular disease, dementia, arthritis, diabetes, osteoporosis, cancers, and infectious diseases (Ali et al., 2008; Kim et al., 2010). The antibacterial activities of gingerols and shogaols have been also studied (Park et al., 2008). More recently, the antibiofilm activities of 6-gingerol (Kim et al., 2015) and 6- and 8-gingerol analogs (Choi et al., 2017) against *P. aeruginosa* have been reported. Interestingly, 6-gingerol inhibited biofilm formation of both *P. aeruginosa* (Kim et al., 2015) (Choi et al., 2017) and *C. albicans* without affecting the planktonic cell growth and showed no chemical toxicity. 6-Gingerol including its analogs interfere the quorum sensing system in *P. aeruginosa*, while 6-gingerol and 6-shogaol suppressed hyphal growth in this study.

Of the six gingerol and shogaol compounds studied in the present study, 6-gingerol and 6-shogaol most effectively reduced *C. albicans* biofilm formation (Figure 1) and 6-shogaol most inhibited biofilm formation, hyphae growth, cell aggregation, and fungal virulence (Figures 1–5). It has been reported on several occasions that the biological potency of 6-shogaol is greater than that of 6-gingerol, and interestingly, these compounds differ structurally by the presence of a hydroxyl moiety in 6-gingerol and double bond on the carbon side chain of 6-shogaol (Figures 1A,D). The presence of this hydroxyl moiety has been previously reported to importantly influence proinflammatory gene activation (Isa et al., 2008). Furthermore, 6-shogaol has been reported to have a markedly stronger anti-tumorigenic effect than 6-gingerol (Wu et al., 2010). Previous studies have suggested a,b-unsaturated carbonyls are susceptible to nucleophilic addition reactions with thiols, such as, glutathione, the most abundant nonprotein thiol *in vivo* (Boyland and Chasseaud, 1968). The transcriptomic analysis conducted in the present study showed 6-gingerol at 50 μg/ml resulted in similar changes in global gene expression as those induced by 6-shogaol at 10 μg/ml (Figure 5), which indicates 6-gingerol and 6-shogaol act at the transcriptional level. We suggest that the structural difference between 6-gingerol and 6-shogaol influence the abilities of these to influence the expressions of hyphae-regulatory genes in the hyphae signaling pathway. Also, we have observed the antibiofilm and antihyphae activities of 8-gingerol (Supplementary Figures S1, S2) and the action mode of 8-gingerol is probably similar to that of 6-gingerol and 6-shogaol in *C. albicans*.

In the present study, we found the transcriptional levels of several hyphae-specific and biofilm-related genes were significantly altered by 6-gingerol and by 6-shogaol (Supplementary Table S1). Gene ontology analysis showed that 6-ginerol and 6-shogaol regulated expression of genes involving membrane components, transport proteins, pathogenesis, stress, and biofilm formation (Supplementary Figure S3). KEGG analysis showed that 6-ginerol and 6-shogaol are similarly associated with several metabolisms such as glycerophospholipid, meiosis, ABC transport, and carbon metabolism (Supplementary Figure S4). Most noticeably, *HWP1* and *ECE1* were down-regulated, and *ECE1* is essential for hyphal development and its expression has been shown to be correlated with cell elongation and biofilm formation (Nobile et al., 2006a). The down-regulations of *HWP1* and *ECE1* by 6-gingerol or 6-shogaol are consistent with their observed effects on biofilm formation and hyphal development. *HWP1* encodes a hyphal wall protein that is essential for hyphal development (Nobile et al., 2006b) and intercellular adherence (Orsi et al., 2014). Previously, we reported that camphor and fenchyl alcohol from cedar leaf oil (Manoharan et al., 2017b) and alizarin from the roots of the madder genus (Manoharan et al., 2017a) inhibit *C. albicans* biofilm formation by reducing hyphal formation by suppressing the gene expressions of *HWP1* and *ECE1*. Thus, it appears that the ability to reduce hyphal formation is not rare in the plant kingdom and that this offers a practical means of inhibiting biofilm formation by *C. albicans*.

On the other hand, both 6-gingerol and 6-shogaol upregulated the expressions of *CDR1* (*Candida* drug resistance, multidrug transporter) and *RTA3* (lipid-translocating exporter) about 10-fold. *CDR1* is a major ABC transporter, and in a previous study, *CDR1* mRNA levels were found to be positively correlated with an increase in azole resistance in *C. albicans* isolates and to be up-regulated during biofilm formation (White, 1997). Ramage et al. reported *CDR1* mutant was highly susceptible to fluconazole when growing planktonically but retained the resistant phenotype during biofilm growth (Ramage et al., 2002). On the other hand, the *RTA3* gene encodes Rta1 p-like lipid-translocating exporter and its expression was found to be positively associated with *CDR1* expression (Whaley et al., 2016). Hence, it is possible that *C. albicans* strives to pump out 6-gingerol and 6-shogaol, which might increase azole-resistance in *C. albicans* when azole antifungal agent(s) and 6-gingerol or 6-shogaol are co-administrated.

The emergence of multidrug resistant *Candida* strains has driven investigations on alternative antifungal agents, and antivirulence and antibiofilm agents have attracted considerable research interest. The present study shows that the antibiofilm effects of 6-gingerol and 6-shogaol on fluconazole-resistant *C. albicans* DAY185 are due to the prevention of yeast-hyphal transition and not to the inhibition of fungal growth. Also, 6-gingerol and 6-shogaol effectively reduced *C. albicans* virulence *in vivo* in a *Caenorhabditis elegans* model with minimal chemical toxicity under the conditions used. In conclusion, 6-gingerol and 6-shogaol have the antibiofilm and antivirulence activities against a drug resistant *C. albicans*.

## Author contributions

J-HL, Y-GK, JGP, and JL designed research, performed experiments, and analyzed the data. PC, JH, and JGP provided materials. J-HL and JL wrote the manuscript. All authors read and approved the final manuscript.

### Conflict of interest statement

The authors declare that the research was conducted in the absence of any commercial or financial relationships that could be construed as a potential conflict of interest.
