# Supplementary material for: Antibiofilm and Antivirulence Activities of 6-Gingerol and 6-Shogaol Against Candida albicans Due to Hyphal Inhibition
Source: Front Cell Infect Microbiol. 2018 Aug 28;8:299. doi: 10.3389/fcimb.2018.00299 (PMC6121036; doi:10.3389/fcimb.2018.00299)

***Supplementary Materials***

**Table S1. Partial list of the common differentially expressed genes of *C. albicans* by 6-gingerol or 6-shogaol.** *C. albicans* cells were grown with and without 6-gingerol (50 µg/ml) or 6-shogaol (10 µg/ml) for 4 h. RNA-seq assays were used to determine differential gene expression.

| **Genes** | | **Descriptions** | **Fold change**  **RNA-seq** | | | | |
| --- | --- | --- | --- | --- | --- | --- | --- |
|  |  |  | 6-Gingerol | | 6-Shogaol | | |
| **Biofilm and hyphae-related proteins** | | | | | | | |
| **ANT1** | Peroxisomal adenine nucleotide transporter | | | -2.2 | | -2.4 | |
| **ARO10** | Aromatic decarboxylase | | | 2.5 | | 1.7 | |
| **ATO5** | Putative fungal-specific transmembrane protein | | | -2 | | -2.1 | |
| **BMT3** | Beta-mannosyltransferase | | | -2.3 | | -2 | |
| **CAT2** | Major carnitine acetyl transferase | | | -2.1 | | -1.8 | |
| **CDR1** | Multidrug transporter of ABC superfamily | | | 7.6 | | 9.9 | |
| **CDR2** | Multidrug transporter, ATP-binding cassette (ABC) superfamily | | | 9.3 | | 15.5 | |
| **CFL2** | Oxidoreductase | | | -2.3 | | -1.7 | |
| **CHT2** | GPI-linked chitinase | | | -2.5 | | -2.9 | |
| **CRP1** | Copper transporter | | | -2.1 | | -2 | |
| **DEF1** | RNA polymerase II regulator | | | -2 | | -2 | |
| **ECE1** | Candidalysin | | | -4.6 | | -2.9 | |
| **FDH1** | Formate dehydrogenase | | | -2.7 | | -2.9 | |
| **FET99** | Multicopper oxidase family protein | | | -2 | | -1.8 | |
| **FGR42** | Protein lacking an ortholog in S. cerevisiae | | | 2.2 | | -1.2 | |
| **FTR2** | High-affinity iron permease | | | -3 | | -2.7 | |
| **GAP2** | General amino acid permease | | | -3.3 | | -2.7 | |
| **HAK1** | Putative potassium transporter | | | -2.5 | | -1.3 | |
| **HWP1(ECE2)** | Hyphal cell wall protein | | | -6.1 | | -7.4 | |
| **IHD1** | GPI-anchored protein | | | -4.3 | | -3.1 | |
| **LDG3** | Putative LDG family protein | | | -2.8 | | -1.4 | |
| **PCL1** | Cyclin homolog | | | -2.2 | | -2.8 | |
| **PCK1** | Phosphoenolpyruvate carboxykinase | | | -6.1 | | -3.2 | |
| **PDR16** | Phosphatidylinositol transfer protein | | | 3.9 | | 5.5 | |
| **PGA10** | GPI anchored membrane protein | | | -2.2 | | -1 | |
| **PGA31** | Cell wall protein | | | -2.2 | | -1.5 | |
| **PGA45** | Putative GPI-anchored cell wall protein | | | -2 | | -1.4 | |
| **PTH2** | Putative cAMP-independent regulatory protein | | | -2 | | -1.3 | |
| **RHD1** | Putative beta-mannosyltransferase | | | -2 | | -1.5 | |
| **RHD3** | GPI-anchored yeast-associated cell wall protein | | | -2.1 | | -2.1 | |
| **RTA3** | Similar to S. cerevisiae Rta1 (role in 7-aminocholesterol resistance) and Rsb1 (flippase) | | | 4.2 | | 6.2 | |
| **SNQ2** | Protein similar to S. cerevisiae Snq2p transporter; member of PDR subfamily of ABC family | | | 3.1 | | 3.7 | |
| **STP4** | C2H2 transcription factor | | | 7.5 | | 10.4 | |
| **TNA1** | Putative nicotinic acid transporter | | | 4.1 | | 3.2 | |
| **TRY4** | C2H2 transcription factor | | | 2.8 | | 4.1 | |
| **UCF1** | Upregulated by cAMP in filamentous growth | | | -2.7 | | -2.4 | |
| **UME6** | Zn(II)2Cys6 transcription factor | | | -2.1 | | -2 | |
| **WOR3** | Transcription factor | | | -2.4 | | -2.6 | |
| **YOR1** | Protein similar to S. cerevisiae Yor1 | | | 3.4 | | 4.4 | |
| **ZSF1** | Ortholog od S. cerevisiae Tis11, a mRNA-binding protein | | | -2.1 | | -2 | |
| **Virulence-related genes** | | | | | | | |
| **FLU1** | Multidrug efflux pump of the plasma membrane | | | 2.1 | | 2.8 | |
| **GIG1** | Protein induced by N-acetylglucosamine (GlcNAc) | | | -3.1 | | -2 | |
| **GIT1** | Glycerophosphoinositol permease | | | -3.1 | | -3.2 | |
| **HGT13** | Predicted sugar transporter, involved in glycerol utilization | | | -2.6 | | -2.7 | |
| **PHO100** | Putative inducible acid phosphatase | | | -2 | | -1.7 | |
| **PLB1** | Phospholipase B | | | -3.3 | | -2.7 | |
| **RME1** | Zinc finger protein | | | -2.3 | | -1.6 | |
| **SAP7** | Pepstatin A-insensitive secreted aspartyl protease | | | -2.7 | | 4 | |
| **Others** | | | | | | | |
| **AMO1** | Putative peroxisomal copper amine oxidase | | | -2.6 | | | -1.3 |
| **BLP1** | Protein of unknown function | | | -2 | | | -3.2 |
| **BTA1** | Ortholog of C. dubliniensis CD36 | | | -3.8 | | | -3.5 |
| **CFL4** | C-terminus similar to ferric reductases | | | -2.1 | | | -2.8 |
| **CFL5** | Ferric reductase | | | -4.4 | | | -3 |
| **DUR3** | High affinity spermidine transporter | | | -2.9 | | | 3.5 |
| **ECM331** | GPI-anchored protein | | | -2.3 | | | -1.7 |
| **EXG2** | GPI-anchored cell wall protein | | | -2.3 | | | -1.8 |
| **FAV1** | Protein with weak similarity to *S. cerevisiae* Fus2p | | | -5.1 | | | -6 |
| **GIT4** | Glycerophosphocholine transporter | | | -3.8 | | | -4 |
| **MFALPHA** | Alpha factor mating pheromone precursor | | | -19.3 | | | -2.3 |
| **PGA58** | Putative GPI-anchored protein | | | -2.5 | | | -2.6 |
| **PHO112** | Putative constitutive acid phosphatase | | | -2.2 | | | -4.4 |
| **ZCF4** | Putative Zn(II)2Cys6 transcription factor | | | -2.8 | | | -1.2 |
| **ZCF5** | Zn(II)2Cys6 transcription factor | | | -2.2 | | | -2 |
| **Hypothetical proteins** | | | | | | | |
| **CAALFM_C100200CA** | Hypothetical protein | | | -2.8 | | | -1.5 |
| **CAALFM_C101220CA** | Hypothetical protein | | | -2.1 | | | -1.7 |
| **CAALFM_C102670CA** | Hypothetical protein | | | -2 | | | 1.7 |
| **CAALFM_C102690CA** | Hypothetical protein | | | -2.5 | | | 1.8 |
| **CAALFM_C103470CA** | Hypothetical protein | | | -4.8 | | | 1.7 |
| **CAALFM_C103880CA** | Hypothetical protein | | | -18.7 | | | -2.3 |
| **CAALFM_C103950CA** | Hypothetical protein | | | -3.4 | | | -1.9 |
| **CAALFM_C104150CA** | Hypothetical protein | | | -2.8 | | | -1.6 |
| **CAALFM_C104270CA** | Hypothetical protein | | | -4.2 | | | -2.1 |
| **CAALFM_C104340CA** | Hypothetical protein | | | -2.2 | | | -1.6 |
| **CAALFM_C104460CA** | Hypothetical protein | | | -2.3 | | | -1.5 |
| **CAALFM_C104690CA** | Hypothetical protein | | | -13.1 | | | -2.5 |
| **CAALFM_C104800CA** | Hypothetical protein | | | -38.4 | | | -2.6 |
| **CAALFM_C104930CA** | Hypothetical protein | | | -4.7 | | | -2.2 |
| **CAALFM_C104940CA** | Hypothetical protein | | | -3.8 | | | -1.7 |
| **CAALFM_C105150CA** | Hypothetical protein | | | -12.8 | | | -13.1 |
| **CAALFM_C105480CA** | Hypothetical protein | | | -2.3 | | | -1.9 |
| **CAALFM_C106620CA** | Hypothetical protein | | | -2.2 | | | 2.4 |
| **CAALFM_C107160CA** | Hypothetical protein | | | -2.4 | | | -1.4 |
| **CAALFM_C109210CA** | Hypothetical protein | | | 2.5 | | | 3.1 |
| **CAALFM_C109500WA** | Hypothetical protein | | | -3.2 | | | -1.5 |
| **CAALFM_C110360CA** | Hypothetical protein | | | 4.4 | | | 5.7 |
| **CAALFM_C111320CA** | Hypothetical protein | | | -2.1 | | | -3.8 |
| **CAALFM_C111390WA** | Hypothetical protein | | | -3.2 | | | -1.3 |
| **CAALFM_C111850WA** | Hypothetical protein | | | 2.1 | | | 2.3 |
| **CAALFM_C112150CA** | Hypothetical protein | | | -2.3 | | | -1.2 |
| **CAALFM_C113100WA** | Hypothetical protein | | | -2.1 | | | -1.9 |
| **CAALFM_C200760CA** | Hypothetical protein | | | 3.7 | | | 4 |
| **CAALFM_C201570WA** | Hypothetical protein | | | 2 | | | 1.6 |
| **CAALFM_C208170WA** | Hypothetical protein | | | 2.1 | | | 1.4 |
| **CAALFM_C210320CA** | Hypothetical protein | | | 2.6 | | | 1 |
| **CAALFM_C301540WA** | Hypothetical protein | | | -8.2 | | | -8.3 |
| **CAALFM_C303460CA** | Hypothetical protein | | | 11.1 | | | 14.3 |
| **CAALFM_C304170WA** | Hypothetical protein | | | 2.3 | | | 2.2 |
| **CAALFM_C305990CA** | Hypothetical protein | | | -6.8 | | | -4.3 |
| **CAALFM_C400990WA** | Hypothetical protein | | | -2.4 | | | -2.1 |
| **CAALFM_C402190CA** | Hypothetical protein | | | 2.3 | | | 1.8 |
| **CAALFM_C403340CA** | Hypothetical protein | | | 9.4 | | | 15 |
| **CAALFM_C403370CA** | Hypothetical protein | | | 2.2 | | | 2.4 |
| **CAALFM_C404190CA** | Hypothetical protein | | | 3.7 | | | 5.1 |
| **CAALFM_C404200CA** | Hypothetical protein | | | 5.1 | | | 7.5 |
| **CAALFM_C405730WA** | Hypothetical protein | | | -2.9 | | | -3.5 |
| **CAALFM_C406200WA** | Hypothetical protein | | | -3.2 | | | -1.3 |
| **CAALFM_C500390CA** | Hypothetical protein | | | -2.1 | | | -1.5 |
| **CAALFM_C502690WA** | Hypothetical protein | | | -2 | | | -1.7 |
| **CAALFM_C503670CA** | Hypothetical protein | | | -2.1 | | | -1.4 |
| **CAALFM_C504010CA** | Hypothetical protein | | | 2.3 | | | 1.8 |
| **CAALFM_C504900CA** | Hypothetical protein | | | 2.1 | | | 1.6 |
| **CAALFM_C504980WA** | Hypothetical protein | | | 2 | | | 1.9 |
| **CAALFM_C600850WA** | Hypothetical protein | | | 2.3 | | | 2.1 |
| **CAALFM_C602100WA** | Hypothetical protein | | | -3 | | | -2.2 |
| **CAALFM_C602200CA** | Hypothetical protein | | | -2.7 | | | -2.8 |
| **CAALFM_C604150WA** | Hypothetical protein | | | 2 | | | -16.5 |
| **CAALFM_C700760CA** | Hypothetical protein | | | 3.6 | | | 2.8 |
| **CAALFM_C700770WA** | Hypothetical protein | | | 8.5 | | | 10.7 |
| **CAALFM_C701380WA** | Hypothetical protein | | | -2.1 | | | -2.2 |
| **CAALFM_C701430CA** | Hypothetical protein | | | 3.5 | | | 3.6 |
| **CAALFM_C702280WA** | Hypothetical protein | | | -5 | | | -1.2 |
| **CAALFM_C704090CA** | Hypothetical protein | | | 2.1 | | | 3.3 |
| **CAALFM_CR01640CA** | Hypothetical protein | | | 2.3 | | | 2 |
| **CAALFM_CR07300WA** | Hypothetical protein | | | -4.6 | | | -2.4 |
| **CAALFM_CR08080WA** | Hypothetical protein | | | 2 | | | 1.4 |

**Table S2: Primer sequences for qRT-PCR**

| Gene | Primer |
| --- | --- |
| *ALS1* | Forward 5'-AGC TGT TGC CAG TGC TTC-3' |
|  | Reverse 5'-AAT GTG TTG GTT GAA GGT GAG-3' |
| *ALS3* | Forward 5'-CAA CAT CAA CCA ACC AAT CTC-3' |
|  | Reverse 5'-TGA ATA ACA GAA CCA GAT CCG-3' |
| *CDR1* | Forward 5'-AGA TGT GTT GGT TCT GTC TCA AAG AC-3' |
|  | Reverse 5'-CCG GAA TAC ATT GAC AAA CCA AG-3' |
| *CDR2* | Forward 5'-CCT GGA AGC ACA GTT GTC CA-3' |
|  | Reverse 5'-TCC CCC TTT TGC ATA GCA CC-3' |
| *CHT2* | Forward 5'-TGA TTT ATT ATC CAA AGT CCC ACT TG-3' |
|  | Reverse 5'-TTG AAT TGG CCA TTG ATT GAA-3' |
| *ECE1* | Forward 5'-CCA GAA ATT GTT GCT CGT GTT GCC A-3' |
|  | Reverse 5'-TCC AGG ACG CCA TCA AAA ACG TTA G-3' |
| *EFG1* | Forward 5’-TAT GCC CCA GCA AAC AAC TG-3’ |
|  | Reverse 5’-TTG TTG TCC TGC TGT CTG TC-3’ |
| *HWP1* | Forward 5'-TTG TTT GCG TCA TCA AGA CTT TG-3' |
|  | Reverse 5'-GTC TTC ATC AGC AGT AAC ACA ACC A-3' |
| *HYR* | Forward 5'-TTG TTT GCG TCA TCA AGA CTT TG-3' |
|  | Reverse 5'-GTC TTC ATC AGC AGT AAC ACA ACC A-3' |
| *PDR16* | Forward 5'-CTG CTG AAC AAC AAG CGA AG-3' |
|  | Reverse 5'-TCG ATT AAA CCC GAG GAT TC-3' |
| *RBT1* | Forward 5'-CTG CCA TTC AAC CAT CTG CTA ACT CCT CAT AC-3' |
|  | Reverse 5'-GCA GCA AGA CCA ATA ATA GCA GCA CCA TAA GT-3' |
| *RTA3* | Forward 5'-CGA AGG CAA ACC AAG TCC AT-3' |
|  | Reverse 5'-TAC CAA TCA TTG CTG CAT CC-3' |
| *SNQ2* | Forward 5'-GCG GAA GAT CGC ACG AAG-3' |
|  | Reverse 5'-GGC GCG AGC GGG ATA-3' |
| *TEC1* | Forward 5'-AGG TTC CCT GGT TTA AGT G-3' |
|  | Reverse 5'-ACT GGT ATG TGT GGG TGA T-3' |
| *UME6* | Forward 5'-AGC ACC AAA TTC GCC TTA TG-3' |
|  | Reverse 5'-AGG TTG AGC TTG CTG CAG TT-3' |
| *RND18* | Forward 5'-AGA AAC GGC TAC CAC ATC CCA-3' |
|  | Reverse 5'-CGA ATG GGC CCT GTA TCG T-3' |

**Figure S1: Microscopic observations of the inhibitory effects of 8-gingerol on biofilm.** Biofilm formation by *C. albicans* on polystyrene plates was observed in the presence of 8-gingerol at 50 µg/ml by confocal laser microscopy (A). Scale bars represent 100 µm. Biofilm formation was quantified by using COMSTAT (B). * = *P*<0.05 *vs.* non-treated controls. None; non-treated control.

**Figure S2: Inhibitions of hyphal filamentation and aggregation by 8-gingerol in liquid medium.** Inhibitions of hyphal filamentation in PDB medium (A) and in RPMI medium (B). *C. albicans* cells were grown for 24 h in PDB medium or RPMI-1640 medium in the absence or presence of 8-gingerol. Hyphae were visualized after incubation for 24 h. The scale bar represents 200 µm. None; non-treated control.

**Figure S3: Gene ontology (GO) distribution of *C. albicans* genes regulated (> 2-fold) by 6-gingerol (A) or 6-shogaol (B) treatment.** The genes were analyzed with the DEG (Differentially Expressed Gene) analysis method in ExDEGA (Excel based Differentially Expressed Gene Analysis) tool and classified by biological processes.


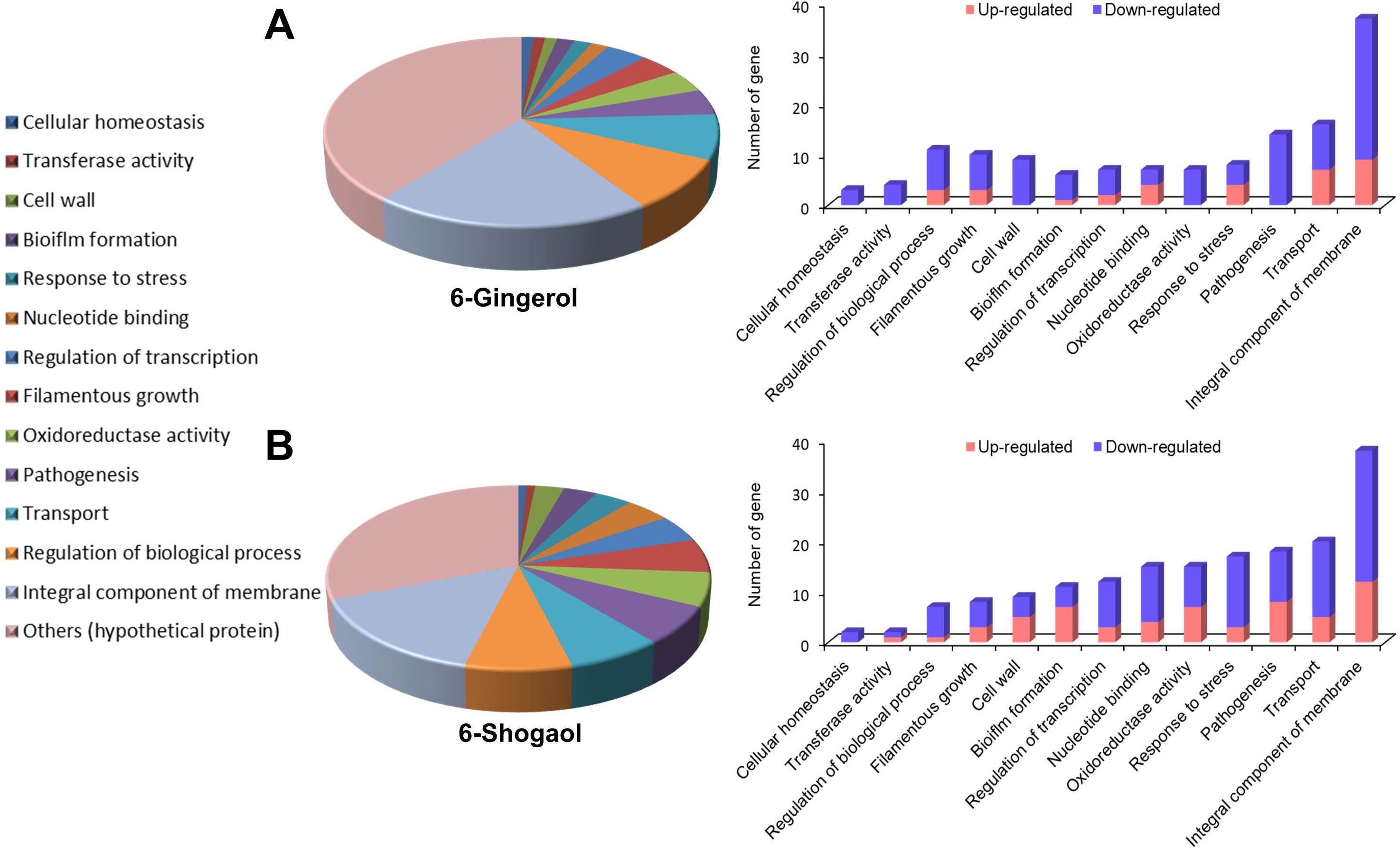


**Figure S4: The KEGG Pathway analysis of the genes up- or down regulated (> 2-fold) by 6-gingerol or 6-shogaol treatment.** The genes were analyzed with the KEGG Mapper tool (<http://www.genome.jp/kegg/tool/map_pathway2.html>). KEGG, Kyoto Encyclopedia of Genes and Genomes.


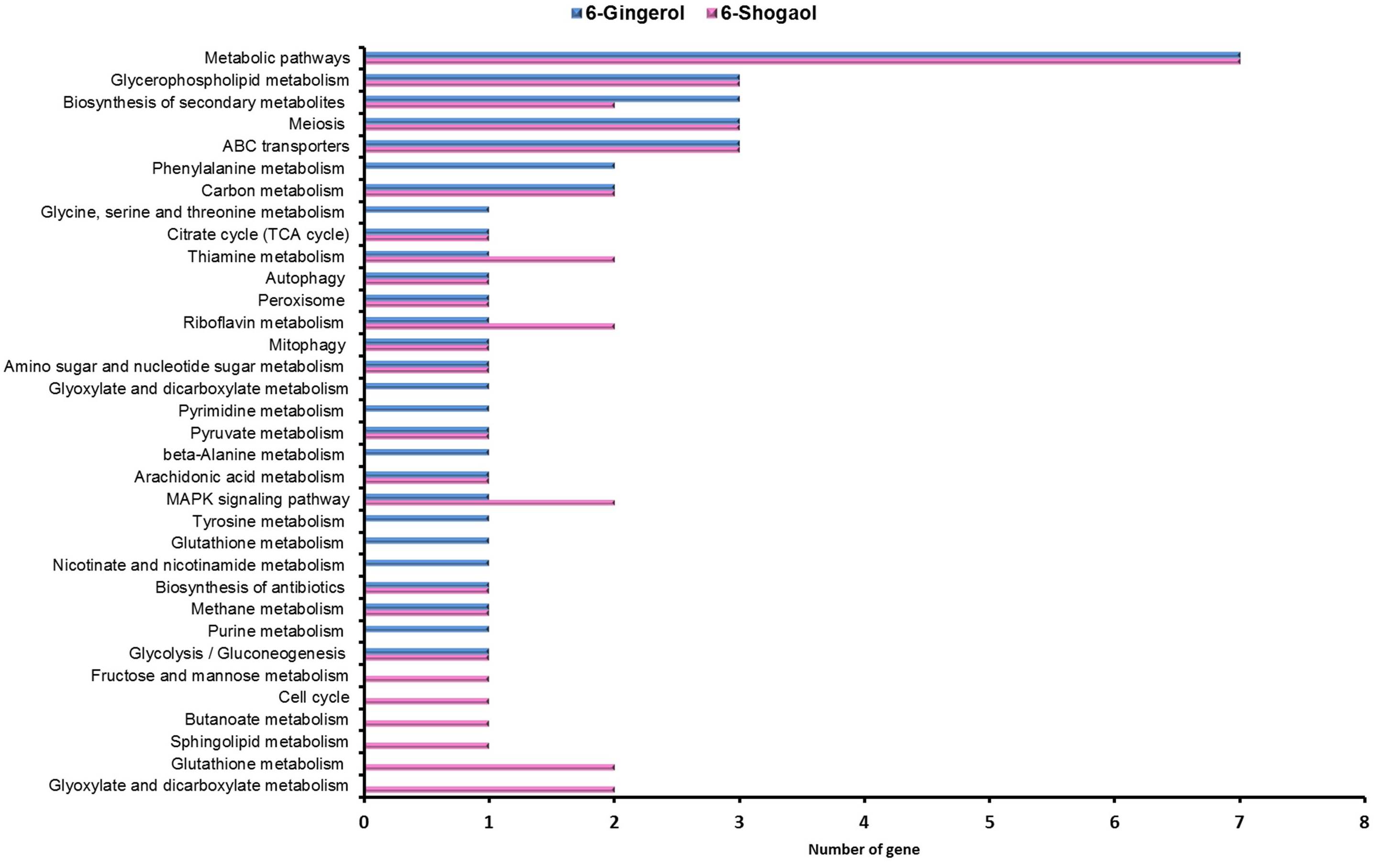

Supplement: Supplementary file 1 [file Data_Sheet_1.doc]
